# An Updated Meta-Analysis of the Efficacy and Safety of Acupuncture Treatment for Cerebral Infarction

**DOI:** 10.1371/journal.pone.0114057

**Published:** 2014-12-01

**Authors:** Li Li, Hong Zhang, Shu-qing Meng, Hai-zhou Qian

**Affiliations:** Department of Neurology, Zhongnan Hospital of Wuhan University, Wuhan, China; St Michael's Hospital, University of Toronto, Canada

## Abstract

**Background:**

Ischemic stroke is the second most common cause of death and the primary cause of disability throughout the world. Acupuncture is frequently advocated as an adjunct treatment during stroke rehabilitation. The aim of this study was to update the clinical efficacy and safety of acupuncture for cerebral infarction.

**Methods:**

Randomized controlled trials (RCT) on acupuncture treating cerebral infarction were searched from the following databases: PubMed, EMBASE, Cochrane Library, CNKI, CMB and VIP from inception to October 2013. The data of RCTs meeting the inclusive criteria were extracted according to Cochrane methods. The meta-analyses were conducted using Rev Man 5.0 software.

**Results:**

A total of 25 trials involving 2224 patients were included. The results of this meta-analysis showed that the groups receiving acupuncture (observation group) were superior to the comparison groups (control group), with significant differences in the Clinical Efficacy Rates [OR = 4.04, 95%CI (2.93, 5.57), P<0.001], Fugl-Meyer Assessment [MD = 11.22, 95%CI (7.62, 14.82), P<0.001], Barthel Index Score [MD = 12.84, 95%CI (9.85, 15.82), P<0.001], and Neurological Deficit Score [MD = −2.71, 95% CI (−3.84, −1.94), P<0.001]. Three trials reported minor adverse events.

**Conclusion:**

Current evidence provisionally demonstrates that acupuncture treatment is superior to either non-acupuncture or conventional therapy for cerebral infarction. Despite this conclusion, given the often low quality of the available trials, further large scale RCTs of better quality are still needed.

## Introduction

Stroke is the second most common cause of death and the leading cause of adult disability worldwide. Due to an aging population, the prevalence of stroke is expected to rise significantly around the world. Currently, clinical therapies for acute cerebral infarction include intravenous thrombolytic, platelet aggregation, improving microcirculation, anticoagulation, neuroprotective agents, and rehabilitation treatment. All these can promote patient's recovery to some extent. After recovery from a stroke, a patient may frequently have motor weakness on one or both sides of the body, which caused great harm to patient's body and mind, meanwhile, it brought heavy economic burden to the family and society. Because the use of complementary and alternative medicine is widespread, and has increased worldwide over the last decade, confirmation of the efficacy of Chinese patent medicine could have a great impact on stroke management [1]–[4].

Acupuncture has been used in traditional Chinese medicine for more than 3000 years as a treatment for many diseases, and is especially well accepted in Asia for rehabilitation after stroke [1]. Sixty-six percent of Chinese doctors use acupuncture for stroke routinely and sixty-three percent believe that it is effective [2]. In 1997, the National Institute of Health Consensus Development Panel on Acupuncture suggested that acupuncture might be a useful adjunct for stroke rehabilitation [3]. Moreover, acupuncture was recommended by the World Health Organization in 2002 specifically for treating stroke patients [4], as it is thought to improve motor, sensation, speech, and other neurological functions. The possible mechanisms of acupuncture's benefits for ischemic stroke patients include facilitation of neural plasticity [5], stimulation of neuronal cell proliferation [6], reduction of excitatory amino acids [7], increased cerebral blood flow, improvement of microcirculation [8] and the inhibition of neuronal apoptosis [9]. Previous systematic reviews [10], [11] of the efficacy of acupuncture for stroke rehabilitation were published in 2010. The results are not consistent. Randomized clinical trials demonstrate that acupuncture may be effective in the treatment of poststroke rehabilitation in one reported [10]. Another meta-analyses of data from rigorous randomized sham-controlled trials did not show a positive effect of acupuncture as a treatment for functional recovery after stroke [10]. Therefore, the aim of this study is to provide an updated systematic review of acupuncture for the treatment of cerebral infarction, and to evaluate the evidence-based medicine for clinical decision.

## Methods

### Eligibility Criteria

In this study, only true randomized controlled trials (RCTs) for evaluating the efficacy of acupuncture on ischemic stroke were included, regardless of blinding, publication status or language. Patients of any age or sex were eligible. Diagnostic criteria were adopted in accordance with the 1995 National Diagnostic Criteria set by the fourth session of cerebrovascular meeting. Diagnosis of ischemic stroke had to be confirmed with CT/MRI scan, and other severe brain diseases were excluded. In the “observation group”, the patients received acupuncture plus rehabilitation or conventional medicine treatment. In the “control group”, the patients had rehabilitation and/or conventional medicine treatment. No crucial differences were found between two groups in the age, sex, duration of disease and neurological deficits before treatment. Inclusion criteria for our systematic review required studies conducted in adult patients (>18 years) with hemiplegia due to ischemic stroke in the acute, subacute or chronic stage were eligible, regardless of time of treatment or the length of the treatment period. Reports had to indicate that patients had to be randomly allocated to either active acupuncture treatment or a control group given sham acupuncture or no acupuncture treatment. Any cointerventions had to be reported as the same in both groups. We excluded studies that reported only laboratory values rather than clinical responses. Comparisons in which acupuncture was not independently assessed were also excluded. The outcome measures were Clinical Efficacy Rate, Fugl-Meyer Assessment [FMA, including Brunnstrom-Fugl-Meyer (BFM) test to determine the level of motor impairment], Barthel Index Score [BI, including the modified Barthel Index (MBI) and a Functional Independence Measure (FIM) to assess the activities of daily living (ADL)], Neurological Deficit Score [NDS, including NHI stroke scale (NIHSS) and Chinese verson of NIH stroke scale (CSS) to evaluate the degree of neurological deficit). Adverse events were reported in three articles at the end of treatment course.

The assessment of the Clinical Efficacy Rate was conducted in accordance with the reduction in the scores of basic nervous functional deficits and disability degree as following: Recovery - The functional deficit scores were decreased up to 91–100%, and disability degree was at grade 0; Remarkable Improvement - The scores of functional deficit were decreased at 46–90%, and disability degree was at the grade 1–3; Improvement - The scores of functional deficit were decreased at 18–45%; No change - The scores of functional deficit were decreased or increased at about 17%; Deterioration - The scores of functional deficit were increased over 18% and death. The FMA scale is a 226-point multi-item Likert-type scale developed as an evaluative measure of recovery from hemiplegic stroke. It is divided into 5 domains: motor function, sensory function, balance, joint range of motion, and joint pain. Each domain contains multiple items, each scored on a 3-point ordinal scale (0 =  cannot perform, 1 =  performs partially, 2 =  performs fully). The motor domain includes items measuring movement, coordination, and reflex action about the shoulder, elbow, forearm, wrist, hand, hip, knee, and ankle. The motor score ranges from 0 (hemiplegia) to a maximum of 100 points (normal motor performance), divided into 66 points for the upper extremity and 34 points for the lower extremity. The BFM test is one of the tests which is applied in order to determine hemiplegia. The test consists of 55 test-items which are scored on an ordinal 3 point scale (0–2 points). The maximum score is 114 points. The original test consisted of testing the upper extremity, lower extremity and balance. The testing of balance however, has no surplus value compared to other assessments that evaluate balance and no surplus value for the results of the BFM. Therefore the test-items for balance are not included in the description of the test and the assessment itself. The maximum score for items assessing motor function is 100 points. The BI is a validated and widely used instrument to measure disability in the ADL. The scores of BI contain bowels, bladder, grooming, toilet use, feeding, transfers (bed to chair and back), mobility, stairs and bathing. The total score was 100. The higher score indicated better independence and poorer dependence. The NIHSS is a 15-item scale that measures the level of neurologic impairment. Total scores on the NIHSS range from 0 to 42, with higher values reflecting more severe cerebral infarcts. The FIM is an 18-item, 7-level functional assessment designed to evaluate the amount of assistance required by a person with a disability to perform basic life activities safely and effectively. It contains 18 items composed of 13 motor tasks and 5 cognitive tasks (considered basic activities of daily living). Tasks are rated on a 7 point ordinal scale that ranges from total assistance (or complete dependence) to complete independence. Scores range from 18 (lowest) to 126 (highest) indicating level of function. The NIHSS is a systematic assessment tool that provides a quantitative measure of stroke-related neurologic deficit. The NIHSS is a 15-item neurologic examination stroke scale used to evaluate the effect of acute cerebral infarction on the levels of consciousness, language, neglect, visual-field loss, extraocular movement, motor strength, ataxia, dysarthria, and sensory loss. A trained observer rates the patent's ability to answer questions and perform activities. Ratings for each item are scored with 3 to 5 grades with 0 as normal, and there is an allowance for untestable items.

### Literature Retrieval

In this meta-analysis, the search of primary articles was conducted in the following databases: PubMed, EMBASE, Cochrane Library, CNKI, CMB and VIP from inception to October 2013. The searches were restricted to either the English or Chinese language. The key search terms in these databases as follows: (1). “cerebral infarction” OR “cerebral ischemia” OR “ischemic stroke” OR “brain infarction” OR “cerebral thrombosis” OR “cerebrovascular disease” OR “brain ischemia”; (2). “acupuncture” OR “acupuncture treatment” OR “acupuncture therapy”; (3). (1) AND (2).

### Study Selection and Data Collection Process

The articles were selected based on the title and abstract in an unblinded standardized manner. The selected articles for all potentially relevant trials were retrieved independently by two authors, and then validated by the other two authors. Disagreements were settled through discussion or consultation with all authors. Two main authors independently collected data on study characteristics (including the first author, patient's condition, observation group, control group, the main points, course of treatment and the main outcomes), into a standardized data extraction form for eligible trials.

### Risk of Bias in Individual Trial

In this review, two main authors assessed the risk of bias of all included trials using the six criteria recommended by the Cochrane Back Review Group [12]. The trials showed low risk bias in selective reporting and incomplete outcome data.

### Synthesis of Results

All statistical analyses were performed by the Rev Man Version 5.0 software. A standard chi-square test and the I^2^ statistic were used to test heterogeneity between trial results. For continuous outcome, weighted mean differences (WMD) or standardized mean differences (SMD) were calculated. For dichotomous outcomes, relative risk(RR)and 95% confidence intervals (CI) were estimated.

## Results

### The Results of Literature Retrieval

**Figure 1:**
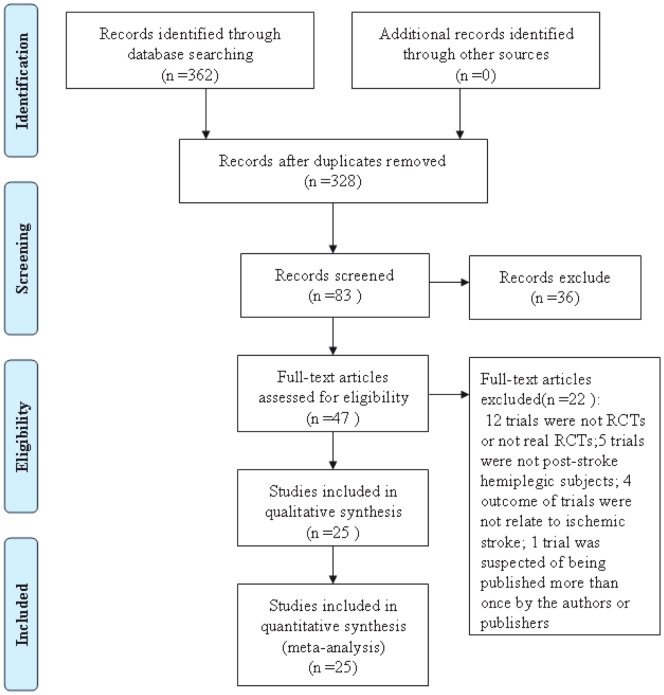
PRISMA 2009 flow diagram.

A total of 362 papers were identified and selected from the databases. Among them, 34 papers were excluded because they were duplicate trials, 245 papers were excluded because they were either case reports, lacked a comparison group, were not clinical trials, or the trials did not focused on acupuncture therapy for cerebral infarction. Of the remaining 47 trials, 12 papers were excluded because they were not RCTs or not real RCTs, 5 papers were excluded because the subjects were not post-stroke hemiplegic, 4 papers were excluded because their outcomes were not related to study, and 1 paper was suspected to have been published more than once. Finally, 25 eligible papers [13]–[37] were identified and included in this updated meta-analysis. The screening process is summarized in a flow diagram (see Figure 1).

In this study, all patients with hemiplegia in the observation group were given acupuncture treatment. Only two trials mentioned the severity of disease [13], [17]. Eighteen trials used manual body acupuncture [13]–[17], [20], [21], [2]–[26], [28]–[31], [33], [35], [36], eight trials used electroacupuncture [18], [19], [22], [27], [32], [34], [37]. Nineteen trials also used scalp acupuncture [13]–[19], [21], [24]–[29], [31], [33], [35]–[37]. Four trials used sham stimulation on acupoints [17], [18], [32], [34]. Seven trials mentioned the starting time when acupuncture was carried out [13], [18]–[20], [27], [28], [33]. The main acupoints were Baihui (GV 20) [14], [16], [24], [26], [29], [33], [35]–[37], Shuigou (DU 26) [15]–[17], [23], [31], Jianyu (LI 5) [13], [15], [19], [21], [22], [24], [25], [37], Quchi (LI 11) [15], [19], [21], [22], [24]–[26], [29], [35], [37], Zusanli (ST 36) [15], [16], [19], [21]–[29], [33], [34], Sanyinjiao (SP 6) [16], [17], [19], [21], [23]–[25], [28], [29], [31], [37], and Hegu (LI 4) [16], [19], [21], [22], [25], [26], [28], [29], [33], [34], [37]. The treatment was conducted once a day by trained doctors (practitoners). The acupuncture retention time varied from 20 minutes [19], [25], [26], [33], [34], [37] to 30 minutes [13]–[16], [18], [20]–[24], [27]–[30], [32], [35]. The total acupuncture treatment lasting for two weeks [15], [16], [22], [33], three weeks [21], [23], [25], [26], [29], or four weeks [13,14.^17^,^19^,^24^,^27^,^28^,^30^–^32^,^34^,^36^,^37^]. See Table 1.

**Table 1:** Characteristics of the included studies.

First author	No. of patients(observation/control)	Observation group	Control group	The main points	Course of treatment	The main outcomes
Tong (2013)[13]	86(44/42)	Acupuncture plus rehabilitation	Rehabilitation	Cuanzhu (BL 2), Dangzhong(CV 17), Jianyu (LI 15), Yanglao (SI 16)	30minutes, once a day, 5 days for a week, 4 weeks	Clinical efficacy Fugl-Meyer scale FIM
Weng (2013)[14]	120(60/60)	Acupuncture plus rehabilitation	Rehabilitation	Penetration needling from Qianshencong (EX-HN 1) to Xuanli (GB 6), penetration needling from Baihui (GV 20) to Qubin(GB 7); Jiquan(HT 1), Chize (LU 5), Biguan (ST 31), Futu (ST 32)	30minutes, once a day, 5 days for a week, 4 weeks	FMA
Du (2013) [15]	60(30/30)	Acupuncture plus western medicine group	Western medicine group	Sibai (ST 2), Shuigou (DU 26), Jianyu (LI 15), Quchi (LI 11), Futu (ST 32), Zusanli (ST 36), Huangshu (KI 16), Tianshu(ST 25)	30–40minutes, once a day, 5 days for a week, 2 weeks	Clinical efficacy NIHSS
Guo (2012) [16]	67(34/33)	Acupuncture plus rehabilitation	Rehabilitation	Baihui (GV 20), Fengchi (GB 20), Shuigou (DU 26), Hegu (LI 4), Neiguan (PC 6), Zusanli (ST 36), Sanyinjiao (SP 6), Taichong(LR 3)	30minutes, once a day, 2 weeks	NIHSS
Shen (2012)[17]	287(144/143)	Acupuncture plus standard treatment	Sham acupoints plus standard treatment	Neiguan (PC 6), Shuigou (DU 26), Sanyinjiao (SP 6), Jiquan (HT 1), Weizhong(BL 40), Chize (LU 5)	Once a day, 4 weeks	NIHSS BI
Hsing	62(35/27)	Scalp		Subcutaneous needles at the projection of the motor,	30minutes,	NIHSS
(2012)[18]		electrical acupuncture stimulation	Placebo electrical stimulation	sensory, frontal and temporal associative areas of penfield homunculus in the scalp	twice a week, 5 weeks	BI
Shu	120(60/60)	Acupuncture	Control group	Jianyu (LI 15), Quchi (LI 11), Shousanli (LI 10),	20minutes, once	BI
(2011)[19]		group		Hegu (LI 4), Xuehai (SP 10), Liangqiu (ST 34), Zusanli (ST 36), Sanyinjiao (SP 6)	a day, 10 treatment for one course, 3 courses	FMA
Cheng	60(30/30)	Acupuncture	Conventional	Zhongzhu (TE 3), Waiguan (TE 5)	30minutes, once	NIHSS
(2011)[20]		plus conventional treatment	treatment		a day, twenty treatment for one course, 2 courses	BI
Wu (2011)[21]	80(40/40)	Acupuncture plus routine	Treatment	Hegu (LI 4), Shousanli(LI 10), Neiguan (PC 6), Waiguan (TE 5), Jianyu (LI 15), Quchi (LI 11); Futu	30minutes, once every other day,	Clinical efficacy NIHSS
		treatment		(ST 32), Zusanli (ST 36), Fenglong (ST 40), Yanglingquan (GB 34), Taichong (LR 3), Sanyinjiao (SP 6), Shenmai (BL 62), Zhaohai (KI 6)	10 times constitute a treatment course, 2 courses	BI
Zhang (2011)[22]	58(29/29)	Electro-needling plus routine treatment	Routine treatment	Jianyu (LI 15), Quchi (LI 11), Shousanli (LI 10), Hegu (LI 4), Biguana (ST 31), Futu (ST 32), Liangqiu (ST 34), Zusanli (ST 36), Fenglong (ST 40), Jiexi (ST 41)	30minutes, once a day, 6 days for a week, 2 weeks	Fugl-Meyer scale
Lin (2009) [23]	56(37/19)	Acupuncture plus western medicine treatment	Western medicine treatment	Shangjuxu (ST 37), Fenglong (ST 40), Zusanli (ST 36), Neiguan (PC 6), Sanyinjiao (SP 6), Shuigou (DU 26)	30minutes, once per day, 3 weeks	Clinical efficacy
Li (2009)[24]	63(31/32)	Acupuncture and routine treatment	Routine treatment	Baihui (GV 20), Lianquan (CV 23) and affected-sided Fengchi (GB 20), Jianyu (LI 15), Quchi (LI 11), Zusanli (ST 36), Sanyinjiao (SP 6) and Taichong (LR 3)	30minutes, once a day, 7 times constitute a course of treatment, 4 weeks	Clinical efficacy
Zhang (2009)[25]	80(40/40)	Acupuncture plus rehabilitation treatment	Rehabilitation treatment	Jianyu (LI 5), Quchi (LI 11), Hegu (LI 4), Yanglingquan (GB 34), Yinglingquan (SP 9), Zusanli(ST 36), Sanyinjiao (SP 6)	20minutes, once a day, 3 weeks	Clinical efficacy Fugl-Meyer scale
Zhang (2008)[26]	90(45/45)	Acupuncture group and	Routine treatment	Scalp motor area (MS 6), sensory area (MS 7), speech area, Baihui(GV 20), Fengchi (GB 20),	20minutes, once a day,	Clinical efficacy NDS
		routine treatment		Quchi (LI 11), Hegu (LI 4), Zusanli (ST 36) and Taichong (LR 3)	10-treatment made up one course, 2 courses	BI
Huang (2008)[27]	80(40/40)	Back-shu point group and routine treatment	Non-point group and routine treatment	Feishu (BL 13), Xinshe (BL 15), Geshu (BL 17), Ganshu (BL 18), Danshu (BL 19), Pishu (BL 20), Wenshu (BL 21), Waiguan (SJ 5), Houxi (SI 3), Zusanli (ST 36), Xuanzhong (GB 39)	30minutes, once a day, 6-treatment for one course, 4 courses	Clinical efficacy BI
Yang (2008)[28]	96(50/46)	Acupuncture plus rehabilitation treatment	Rehabilitation treatment	Qihai (CV 6), Guanyuan (CV 4), Hegu (LI 4), Zusanli (ST 36), Sanyinjiao (SP 6) and Xuehai (SP 10)	30minutes, once a day, 1-month treatment made up a course	Clinical efficacy
Guo (2007)[29]	88(46/42)	Acupuncture plus routine treatment	Routine treatment	Baihui (GV 20) penetrating to Qubin (GB 7) on the affected side, Renzhong (GV 26), Lianquan (CV 23), Neiguan (PC 6), Quchi(LI 11), Hegu (LI 4), Xuehai (SP 10), Zusanli(ST 36), Fenglong (ST 40), Sanyinjiao (SP 6) and Taichong (LR 3)	30minutes, once a day, 5 times a week, 3 weeks	Clinical efficacy
Feng (2007)[30]	60(30/30)	Acupuncture plus routine treatment	Routine treatment	Bilateral Fengchi (GB 20), Wangu (GB 12) and Tianzhu (BL 10)	30minutes, once a day, 15 days as a course of treatment, 2 course	Clinical efficacy
Rao (2006)[31]	40(20/20)	Acupuncture plus routine treatment	Routine treatment	Neiguan (PC 6), Shuigou (DU 26), Sanyinjiao (SP 6)	Once a day, 5 days for a week, 4 weeks	BI
Schuler (2005)[32]	81(41/40)	Acupuncture group	Placebo group(surface electrodes on acupuncture points with visual stimulation)	Needling of acupuncture points with electrical stimulation	30minutes, twice weekly for 4 weeks	BI
Zhu (2005)[33]	80(40/40)	Acupuncture plus routine treatment	Routine treatment	Baihui (GV 20), Fengchi (GB 20), Jiquan (HT 1), Neiguan (PC 6), Hegu (LI 4), Zusanli (ST 36), Taichong (LR 3), Taixi (KI 3), Fuliu (KI 7), Yinbai (SP 1) and Zuqiaoyin (GB 44)	20minutes, once a day, 12 days	Clinical efficacy NDS
Xie	64(32/32)	Electro-acupuncture	Placebo	Yuji (LU 10), Hegu (LI 4), Waiguan (SJ 5),	10–20minutes,	NIHSS
(2004)[34]			needle	Shousanli (LI 10), Yanglinquan (GB 34), Zusanli	once a day,	FMA
		treatment	treatment	(ST 36), Xuanzhong (GB 39), Taichong(LR 3)	7–10days for one course, 2–3 courses	MBI
Liu (2001)[35]	160(120/40)	Acupuncture plus routine treatment	Routine treatment	Baihui (GV 20), Dazhui (DU 14), Zusanli (ST 36), Shousanli (LI 10), Xuehai (SP 10), Quchi (LI 11)	30minutes, once a day, 6 days for a week, 2 months	Clinical efficacy
Wu (2001) [36]	100(50/50)	Scalp acupuncture treatment	Medication treatment	A needle was inserted into Baihui (GV 20) then advanced to Qianding (GV 21), another needle was penetrated from Shuaigu (GB 8) to Xuanli (GB 6)	Once a day, 14 days for one course, 2 courses	Clinical efficacy
Pei	86(43/43)	Electro-acupuncture	Routine	Hegu (LI 4), Shousanli (LI 10), Quchi (LI 11),	20minutes, once	CSS
(2001)[37]		plus	treatment	Jianyu (LI 15), Sanyinjiao (SP 6), Fenglong (ST 40),	a day, 5 days for	BMF
		routine treatment		Zusanli (ST 36), Baihui(DU 20) and motor area of the scalp	a week, 4 weeks	BI

FMA: Fugl-Meyer Assessment, BFM: Brunnstrom-Fugl-Meyer (BFM test for determining the level of motor impairment), BI: Barthel Index, FIM: Functional independence measure, MBI: Modified Barthel Index, NIHSS: NHI Stroke Scale, DNS: Neurological Deficits Score, CSS: Chinese Stroke Scale(CSS for assessing the degree of neurological deficit).

### Characteristics of Included Trials

The 25 randomized clinical trials included 2224 individual patients. There were 1171 patients in the “observation groups” and 1053 patients in the “control groups”. The outcomes included the Clinical Efficacy Rate, motor function assessment, Barthel index scores and neurological deficit scales. Clinical Efficacy Rates were reported in 14 papers [13], [15], [21], [23]–[30], [33], [35], [36], Fugl-Meyer Assessment Scores were assessed in 7 papers [13], [14], [19], [22], [25], [34], [37], Barthel Index Scores were measured in 12 papers [13], [17]–[21], [26], [27], [31], [32], [34], [37], and Neurological Deficit Scores were calculated in 10 papers [15]–[18], [20], [21], [26], [33], [34], [37]. The detailed characteristics of the included papers are listed in Table 1.

### The Study of Quality Evaluation

Among 25 RCTs trials, the baselines of patients were reported. Only three papers [16], [17], [24] explicitly reported the method of random sequences generation, two papers [17], [31] also described concealment of allocation, and five papers [17], [18], [27], [32], [34] referred to blinding procedures. The methodological quality of each trial is described in Table 2. The present study does not have an increased risk of bias.

**Table 2:** Cochrane risk of bias results.

Studies	Adequate sequence generation?	Allocation Concealment?	Free of selective reporting?	Free of other bias?	Blinding?	Incomplete outcome data addressed?
Tong [13]	U	N	Y	U	U	Y
Weng [14]	U	N	Y	U	U	Y
Du [15]	U	N	Y	U	U	Y
Guo [16]	Y	N	Y	U	U	Y
Shen [17]	Y	Y	Y	U	Y	Y
Hsing [18]	U	N	Y	U	Y	Y
Shu [19]	U	N	Y	U	U	Y
Cheng [20]	U	N	Y	U	U	Y
Wu [21]	U	N	Y	U	U	Y
Zhang [22]	U	N	Y	U	U	Y
Lin [23]	U	N	Y	U	U	Y
Li [24]	Y	N	Y	U	U	Y
Zhang [25]	U	N	Y	U	U	Y
Zhang [26]	U	N	Y	U	U	Y
Huang [27]	U	N	Y	U	Y	Y
Yang [28]	U	N	Y	U	U	Y
Guo [29]	U	N	Y	U	U	Y
Feng [30]	U	N	Y	U	U	Y
Rao [31]	U	Y	Y	U	U	Y
Schuler [32]	U	N	Y	U	Y	Y
Zhu [33]	U	N	Y	U	U	Y
Xie [34]	U	N	Y	U	Y	Y
Liu [35]	U	N	Y	U	U	Y
Wu [36]	U	N	Y	U	U	Y
Pei [37]	U	N	Y	U	U	Y

Y: yes (low risk bias); N: no (high risk bias); U: unclear.

### Outcome Measures

**Figure 2:**
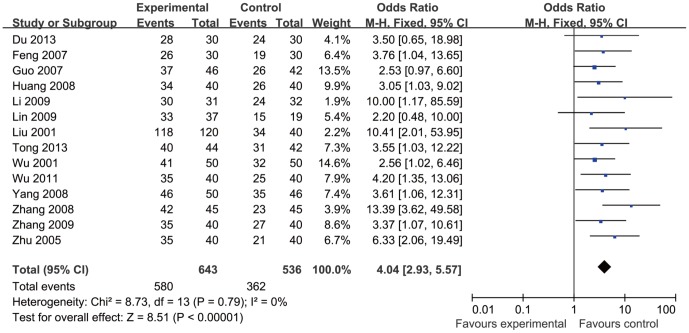
Forest plot showed Clinical Efficacy Rate of acupuncture treatment for cerebral infarction.

Clinical Efficacy Rates were reported in 14 papers [13], [15], [21], [23]–[30], [33], [35], [36], and showed homogeneity in the consistency of the trial results (heterogeneity: chi-square  = 8.73, P = 0.79; I^2^ = 0%). The combined results showed the Clinical Cfficacy Rate improved significantly in acupuncture therapy (observation group) when compared with the “control group” (OR = 4.04, 95% CI: 2.93–5.57, P<0.001), see Figure 2.

**Figure 3:**
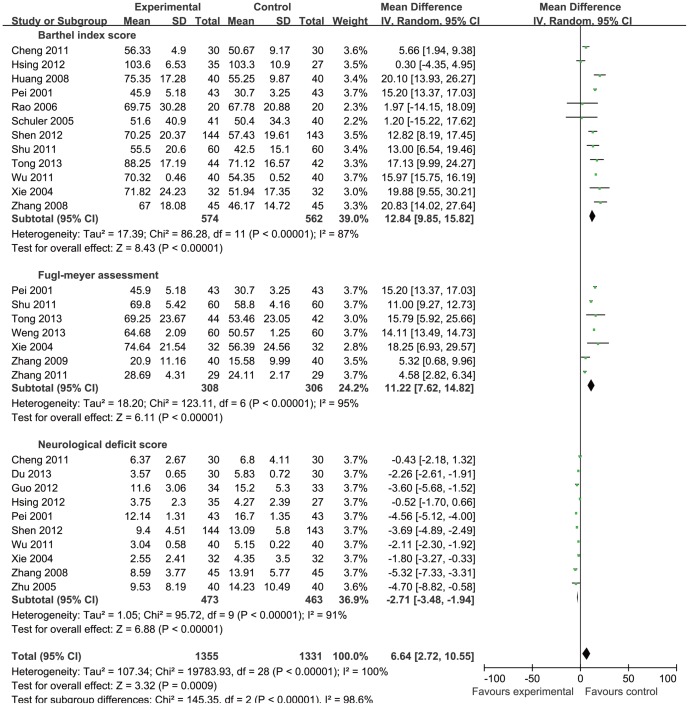
Forest plot showed Barthel Index Score, Fugl-Meyer Assessment and Neurological Deficit Score of acupuncture treatment for cerebral infarction.

Barthel Index Scores were reported in 12 papers [13], [17]–[21], [26], [27], [31], [32], [34], [37], and showed heterogeneity in the consistency of the trial results (heterogeneity: chi-square  = 86.28, P<0.001, I^2^ = 87%). The combined results demonstrated that the Barthel Index Scores improved significantly in acupuncture therapy (observation group) when compared with the “control group” (MD = 12.84, 95% CI: 9.85–15.82, P<0.001), see Figure 3.

Fugl-Meyer Assessment was reported in 7 papers [13], [14], [19], [22], [24], [25], [27], and showed heterogeneity in the consistency of the trial results (heterogeneity: chi-square  = 123.11, P<0.001, I^2^ = 95%). The combined results showed the Fugl-Meyer Assessment Scores improved significantly in acupuncture therapy (observation group) when compared with the “control group” (MD = 11.22, 95% CI: 7.62–14.82, P<0.001), see Figure 3.

Neurological Deficit Scores were reported in 10 papers [15]–[18], [20], [21], [26], [33], [34], [37], and showed heterogeneity in the consistency of the trial results (heterogeneity: chi-square  = 95.72, P<0.001, I^2^ = 91%). The combined results showed the neurological deficit scores improved significantly in acupuncture therapy(observation group)when compared with the “control group” (MD = −2.71, 95% CI: −3.48 to −1.94, P<0.001), see Figure 3.

Adverse Events were reported in three papers. One paper [17] reported that one patient had transient fainting, ten patients had pain at the insertion site, and one patient had blood pressure evaluated during acupuncture treatment. A second study [27] reported that two patients experienced dizziness during acupuncture treatment. A third paper [32] found three patients had superficial hematoma around the acupuncture points.

## Discussion

### Evaluation the Methodology of the Trials

Acupuncture treatment is thought to be a highly feasible intervention for stroke patients. The double blind method does not conform to the clinical practice both for the practitioners and the patients' use. Thus, in the process of random distribution and outcome measurement using the blind method is particularly important. This meta-analysis included 25 trials, although they all reported that they utilized clinical randomized control procedures, only three trials described the method of random sequences generation [16], [17], [24], while two trials described the hidden method [17], [31]. Thus, it is difficult to determine the quality and authenticity of the RCT methods. Five trials mentioned the blinding of assessment [17], [18], [27], [32], [34], four trials referred to the follow-up [17], [31], [32], [37], the other trials did not mention if patients withdrew or were lost to follow-up. In order to improve and consolidate the clinical efficacy, it deserves further exploration and research.

### Summary of Therapy Efficacy

Previous systematic reviews of the efficacy of acupuncture for stroke rehabilitation were published in 2010 with inconsistent results [10], [11]. To evaluated the clinical efficacy of acupuncture in adult patients with disability after stroke. The 56 randomized clinical trials included 5650 patients, 3156 in the treatment groups and 2494 in the control groups. Classified based on cause of stroke, 4 trials assessed hemorrhagic stroke, 24 trials assessed ischemic stroke and 28 trials assessed either hemorrhagic or ischemic stroke (mixed). Classified on the basis of acupuncture administered, 16 studies used electro- acupuncture, 24 studies used both scalp and body acupuncture, 28 studies used body acupuncture, and 4 studies used scalp acupuncture only. Sham acupuncture was used as control in 7 studies, the remaining 41 studies did not use any type of sham acupuncture as a control. Physical rehabilitation, conventional medication, or traditional Chinese medicines were used equally in both intervention and control groups. Forty-six studies were conducted in patients whose acupuncture was given within acute status (1 month after stroke). Ten studies tested the use of acupuncture in disability (0.8 to 24 years poststroke). The intervention duration ranged from 2 to 10 weeks. The results showed that acupuncture may be effective in the treatment of poststroke rehabilitation [11].

Another study identified 664 potentially relevant ten articles, involving a total of 711 participants [10]. Seven trials included patients in the acute or subacute stage of stroke, and three included patients in the chronic stage. Eight trials were from Western countries and published in English. Two trials were from China and published in Chinese. Two trials used sham electro-stimulation on acupoints. The other eight used sham acupuncture:one trial used superficial acupuncture in the control procedure; four used nonpenetrating acupuncture on nonacupuncture points or real acupuncture points; and three used penetrating acupuncture on nonacupuncture points. Four trials tested the effects of acupuncture on neurologic deficits using the NIHSS, the European Stroke Scale or the Scandinavian Stroke Scale. One of the four studies showed positive therapeutic effects. Seven trials tested the effects of acupuncture on activities of daily living according to the Barthel Index, the modified Barthel Index or the Sunaas Index of Activities of Daily Living. Two of these studies showed favourable effects on activities of daily living. Of the five trials that assessed the effects of acupuncture treatment on quality of life, none showed favourable effects. Two of the seven trials that involved patients in the acute and subacute stages of stroke were excluded from the meta analysis. The meta-analysis of the remaining five studies did not yield a significant difference in favour of acupuncture. The same findings applied to ADL after more than six months of follow-up measured with the BI. For global neurologic deficits, none of the three trials included in the meta-analysis showed a significant difference in favour of acupuncture. Of the three trials that involved patients in the chronic tage of stroke, one was excluded from the meta-analysis ecause it did not report numerical data. A meta-analysis of the data from the other two studies did not show favourable ffects of acupuncture on function according to the Modified shworth Scale.

In this study, a total of 25 trials with 2224 participants suffering from cerebral infarction were selected for meta-analysis. The main findings were that adjunct acupuncture therapy significantly improved the Clinical Efficacy Rate, Fugl-Meyer assessment scores, Barthel index scores and neurological deficit scores in patients with cerebral infarction when compared to conventional treatment (control group). This suggests that patients may benefit from acupuncture treatment after cerebral infarction. This is consistently with other meta-analyses showing that “scalp” acupuncture significantly improves neurological deficit scores and the clinical effectiveness rates when compared with conventional, western treatments [38].

As the brain suffers ischemic damage, endogenous neural stem cells are activated, and prime the ischemic brain to promote the survival of the newly formed progenitor cells [39]. Acupuncture stimulates cells proliferation and the differentiation of astroglia into mature neurons [40]. In an animal study, a significant difference was found in the scores of rat neurological deficits between the electroacupuncture and control groups 7, 14 and 21 days after cerebral ischemic injury [40]. A large number of clinical observation and experimental studies also confirm that acupuncture therapy in human and animal models of cerebral infarction have many positive effects. Acupuncture at acupoints ST 36 (Zusanli) and LI 4(Hegu)activates descending antinociceptive pathway and deactivates multiple limbic areas associated with pain systems [41], [42]. Functional MRI studies also demonstrate multiple activation sites in the periphery of the ischemic area following acupuncture [43].

### Limitations and implications

This meta-analysis has several potential limitations that should be taken into account. First, only a few trials explicitly described the method of random distribution, allocation concealment and blind assessment. Second, most studies never mentioned patients who were lost to follow-up. Third, the sample populations were small, with more than 100 cases reported in only four of the trials [14], [17], [19], [35]. Fourth, the amount of time for acupuncture treatment is not consistent across studies. The shortest time reported was 2 weeks, and the longest time was 6 months. Fifth, trials used varying acupuncture protocols (such as needle type, needle stimulation, the depth of insertion), and the different practitioners had varying backgrounds. Varied acupuncture time is a case of varied acupuncture. As almost all included papers are of Chinese origin and only a minority published in Western journals. This may cause a small but possible risk of bias. Future clinical trials should attempt to standardized the acupuncture protocol, utilize skilled practitioners, and utilize consistent outcome measures. Although acupuncture therapy is generally considered safe and well tolerated, future trials should also pay more attention to its safety.

In conclusion, the results of this systematic review shows that acupuncture therapy is significantly effective in improving the Clinical Efficacy Rate, Fugl-Meyer Assessment Scores, Barthel Index Scores and Neurological Deficit Scores in ischemic stroke patients when compared with the conventional medication. Further large-scale, well-designed RCTs on this topic are still warranted.

## Supporting Information

Checklist S1 **PRISMA 2009 checklist.**
(DOC)
